# Effect of Different Crown Materials on the InterLeukin-One Beta Content of Gingival Crevicular Fluid in Endodontically Treated Molars: An Original Research

**DOI:** 10.7759/cureus.1361

**Published:** 2017-06-16

**Authors:** Prathibha Saravanakumar, Padmanabhan Thallam veeravalli, Anand Kumar V, Kasim Mohamed, Umamaheswari Mani, Manita Grover, Saravanan Thirumalai Thangarajan

**Affiliations:** 1 Department of Prosthodontics, Faculty of Dental Sciences, Sri Ramachandra University, Porur, Chennai, India; 2 Prosthodontics, MN Dav,Tatul,Solan,Himachal Pradesh

**Keywords:** interleukin 1 beta, endodontically treated molars, inflammation, metal crowns, ceramic crowns, zirconia crowns

## Abstract

**Introduction:**

Crown materials used in fixed prosthodontics come into close and prolonged contact with the gingiva.

**Objective:**

The purpose of this study was to evaluate the effect of different crown materials on the interleukin-one beta (IL-1β) content of the gingival crevicular fluid and to study which crown material causes the highest inflammation on the marginal gingiva on a biochemical basis.

**Materials and Methods:**

Twenty patients with single endodontically treated tooth were examined. Contralateral teeth were taken as controls. The crown materials in contact with the marginal gingiva were divided into three groups: Group 1- metal, Group2- ceramic, Group 3-zirconia. The collected data were analyzed with International Bibliography of the Social Sciences (IBSS). Statistical Package for the Social Sciences (SPSS) Statistics software 23.0 (IBM Corp, Armonk, New York). All assay procedures were carried out and the results of the collected samples were calculated using the ELISA-AID^TM^ technique.

**Results:**

Multiple comparisons using one-way analysis of variance (ANOVA) between the materials on day zero, 45^th ^and 90^th^ day was highly significant with p=0.0005. Pairwise comparison using Tukey’s honest significant difference (HSD) posthoc test was also highly statistically significant with p= 0.0005 except for ceramic & zirconia which were significant at p=0.04 on the 90^th^ day. Multiple comparison using repeated measure of ANOVA with Bonferroni correction between day zero, 45^th^ and 90^th^ day was found to be statistically significant only for zirconia (p=0.002).

**Conclusion:**

This study was conducted to evaluate the effect of different crown materials on the amount of marginal gingival inflammation by measuring the IL-1β content in gingival crevicular fluid (GCF). At the end of the three-month analysis, it was seen that the zirconia crowns exhibited the least marginal gingival inflammation.

## Introduction

The main objectives of fixed dental prosthesis include replacement of missing natural teeth and to establish and maintain periodontal health. Other objectives include maintenance of the form and function, prevention of residual root fracture, aesthetics and retention of the final restoration. Different crown materials along with margin placement and pontic design affect the health of the periodontium.

When selecting a dental casting alloy for a clinical situation, the dentist’s decision may be influenced by the physical properties of the alloys, cost, and biocompatibility [1]. Dental casting alloys vary differently in composition and some of them contain toxic elements, such as nickel, cobalt, lead, cadmium, and beryllium. Certain dental alloys tend to cause gingival and periodontal inflammation and if not identified early, they can lead to periodontal breakdown and further material failure. Despite the long-standing use of alloys and ceramic as fixed and removable restoration materials, there still are open questions about their behavior in the biological environment [2].

Metal ceramic systems combine both the exceptional esthetic properties of ceramics and the extraordinary mechanical properties of metals. Some metals used as restorative materials in dentistry may constitute a problem for some patients. The drawbacks, as well as the search for more esthetic materials by patients and dentists, have stimulated research and development of metal-free ceramic systems [3].

Since alloys used in dentistry come into close and prolonged contact with the gingiva and oral mucosa, prosthodontic research must involve cellular and molecular biological approaches to assess the host’s immune status and chronic inflammatory responses to the materials in contact with the oral tissues.

Gingival crevicular fluid (GCF) is often attracted as a marker of periodontal disease activity. It is an inflammatory exudate that can be collected at the gingival margin or gingival crevice. Host response in periodontal disease can be assessed non-invasively by the biochemical analysis of GCF [4]. The interleukin one beta (IL-1β) is a potent inflammatory cytokine, since it recruits neutrophils to the inflamed site, being generally induced by bacterial antigens. Additionally, it has been suggested and used as one of the main markers of acute inflammation. [5]

The collection of GCF may provide information on fluid volume and flow rate in clinical assessments of gingival inflammation.

This study was conducted to evaluate the effect of different crown materials on the IL-1β content of the gingival crevicular fluid and to assess which crown material causes the least inflammation of the marginal gingiva.

## Materials and methods

This study was an in vivo methodology done in collaboration with the department of endodontics and department of biochemistry (biochemical analysis) in Sri Ramachandra University, from July to September for a time period of three months. Twenty patients; 10 males and 10 females in the age group of 20-40 years of age (with a mean age of 30) and with single endodontically treated molars (maxillary/mandibular) were included in the study. The subjects rights were protected by the institutional board and written informed consent was granted by all subjects. The endodontically treated molar tooth was selected for the study since these are the tooth which is to receive mandatory crowns after root canal treatment. Only cases with adjacent and contralateral teeth present (which were taken as controls) were considered. The study subjects were divided such that 10 patients received metal ceramic crowns and 10 patients received zirconia crowns. The ten patients who received the metal ceramic crowns were again divided into two groups; group one-metal (Bella Bond N, Germany) and group two-ceramic (Dentsply, Ceramco, U.S.A) such that the buccal sulcular region is in contact with the ceramic portion of the crown and the lingual sulcular region is in contact with the metal portion of the crown. The patients with diabetes mellitus, hypertension, gingivitis or periodontal disease which has an impact on the GCF levels were not included in the study. To allow the inflammation present after endodontic treatment to subside, a rest period of 10 days was given to the patients before crown preparation and sample collection. The patients considered in this study also underwent professional scaling one week prior to the crown preparation and were instructed to follow routine oral hygiene procedures followed by rinsing with 0.12% chlorhexidine (15 ml for 30 seconds, twice a day). Patients were instructed to use mouth rinse throughout the study period in order to maintain gingival health. Informed consent was obtained from the patients and all the procedures carried out were in accordance with the ethical standards.

A questionnaire (Table 1) was prepared and the details were recorded prior to tooth preparation on endodontically treated tooth.

**Table 1:** Questionnaire given to the patients prior to tooth preparation

S.no	Questionnaire	Answers
1	Name and age of the patient	
2.	Was posterior tooth root canal treated and which tooth	Yes/No
3.	Do you have any medical complications?	Yes/no
4.	Was professional scaling procedure carried out one week prior to crown preparation as instructed?	Yes/no
5.	Are you rinsing the mouth with mouthwash twice daily as instructed (0.12% chlorhexidine, 15 ml for 30 seconds, twice a day)?	Yes/no
6.	Were you comfortable when the root canal treatment was carried out?	Yes/no

Tooth preparation in endodontically treated teeth in the study was carried out starting with depth orientation grooves made with round end tapered bur (TR-14, Mani diamond rotary instruments (ISO 198/022),Tochigi, Japan) along the cuspal inclines. A reduction of 1.5 mm to 2.0 mm was carried out. To maintain the standard, burs were changed after every two preparations (two patients).

**Figure 1:**
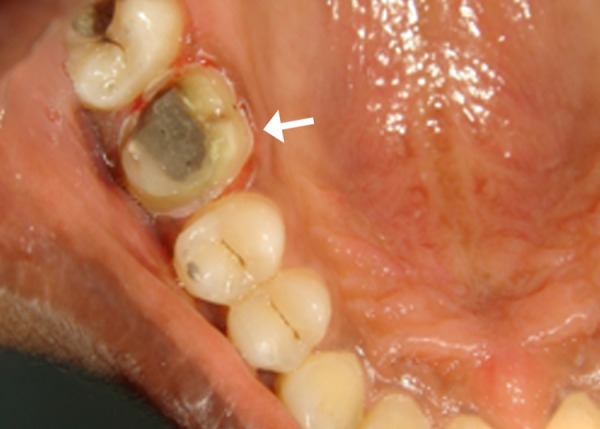
Figure showing the crown prepared with equi-gingival finish line

Care was taken to place the margins equi-gingivally and that no bleeding was induced due to margin placement (Figure 1).

A shoulder finish line configuration was prepared for the buccal aspect of the metal ceramic crowns and chamfer finish line for the ceramic part. Similarly, a chamfer finish line was advocated for the teeth which were to receive zirconia crowns. According to manufacturer’s recommendations, polyvinyl siloxane putty material (Aquasil Soft Putty/Regular set) was manipulated and loaded onto the tray. Wash material quasil light body (Dentsply International, Pennsylvania, United States) was syringed around the preparation and putty material pressed over the preparation. No gingival retraction cord was used for any of the patients to prevent bleeding. The impression was then poured in die stone (Elite rock, Zhermack technical, Italy).

Provisional restorations (Protemp plus temporization material, 3M, Minnesota, United States) were fabricated and luted with zinc oxide eugenol cement (Dentsply, Detray, Germany) for all the patients. Within one-week, permanent crowns were fabricated and cemented with the permanent luting agent (GC Corporation Tokyo, Japan). Three different crown materials were used in the study.

Group1: nickel-chrome metal-to-ceramic alloy (Bellabond N, Germany)

Group2: ceramic (Dentsply, Ceramco, U.S.A),

Group3: zirconia (IPS D-sign)

The total duration of the study was 90 days and the samples were collected from the patients on day zero, 45th and 90th day.

The first sample collection (GCF collection) was done immediately after crown placement and was considered as the day zero for the study.

**Figure 2:**
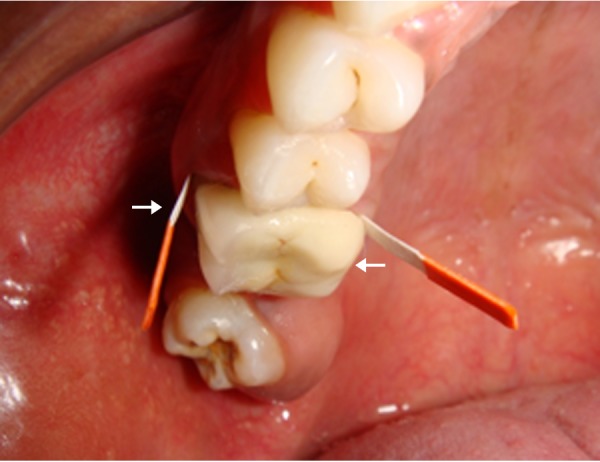
Figure showing the gingival crevicular fluid (GCF) samples collected on the tooth

The site was prepared for sample collection by removing supragingival plaque, isolating with cotton rolls and gently air drying the site. GCF was collected with the help of prefabricated 2 x 13-mm PerioPaper strips (Ora flow Inc, New York, U.S.A), which were inserted into the gingival crevice until mild resistance was felt, taking care to avoid mechanical trauma. The strips were placed into the buccal crevice region on the ceramic facing aspect of the crowns and inserted into the lingual crevice region where the metal margin comes into contact with the marginal gingival (Figure 2).

**Figure 3:**
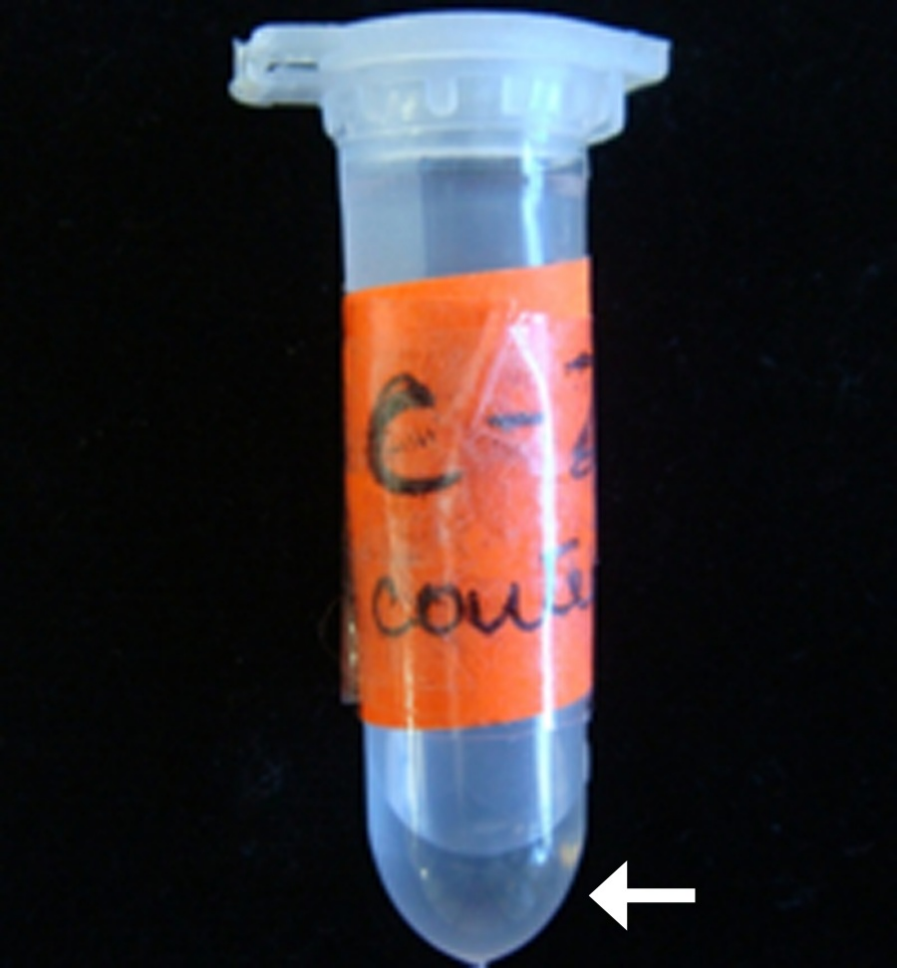
The periopaper strips with the collected gingival crevicular fluid (GCF) stored in the labelled eppendorf tubes with storage medium as trish-Hcl

This was done for the contralateral natural tooth also. The strips were left in site for 30 seconds and were then removed with the help of a tweezer and stored in Eppendorf tubes containing 0.5 ml of trisaminomethane hydrochloride (Tris-Hcl ) until further processing (Figure 3).

Samples contaminated with blood were discarded. Similar samples were collected on the 45th and 90th day of the test site also. GCF was eluted from the paper strips by incubation for 30 minutes. The elution was carried out with the help of a cyclomixer (Remi Electrotechnik Limited, Thane, India). Cyclomixer is a variable speed mixer which is used to eliminate time-consuming hand mixing, and its speed regulator controls the degree of vibration. The Eppendorf tube was held against the vibrating rubber cup to allow rapid mixing of contents. After elution, the paper strip was removed and the eluate was centrifuged for minutes and stored frozen at -70 degrees centigrade for later assay.

**Figure 4:**
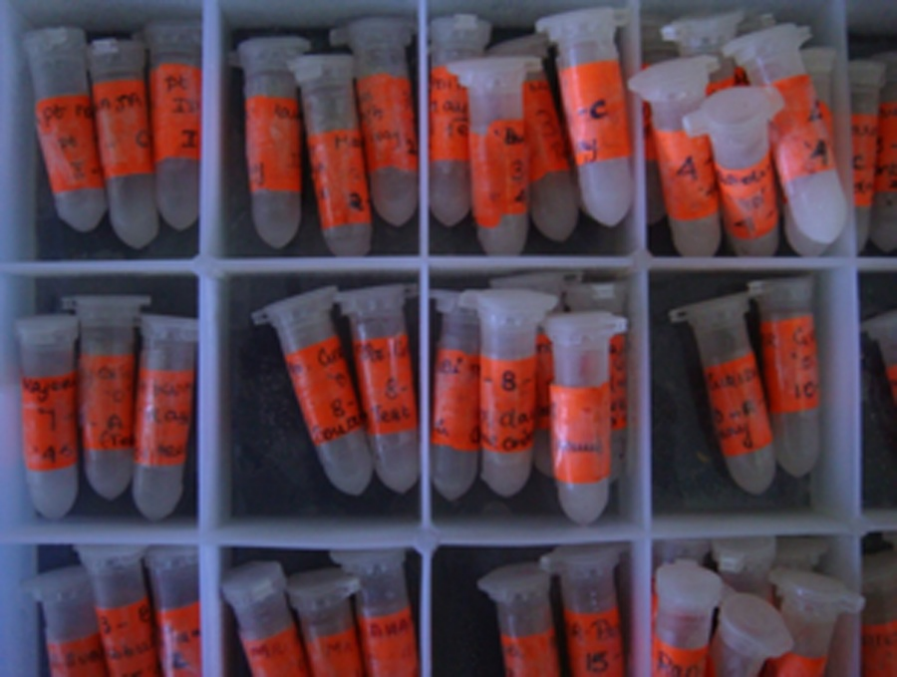
The vortexed samples placed in sectioned plastic containers

The samples were stored at -70 degrees centigrade in sectioned plastic containers and placed in ultra low freezers (Figure 4).

**Figure 5:**
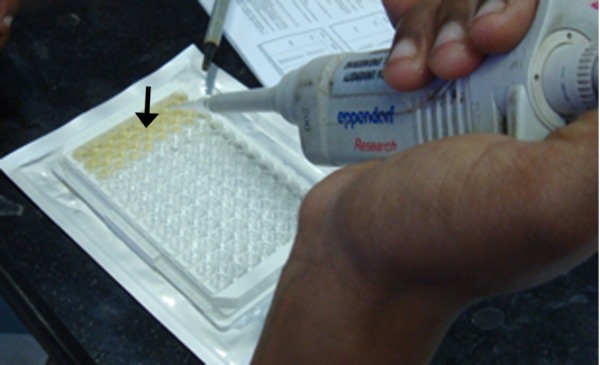
Figure showing samples and controls added

The DIA source IL-1β- EASIA Kit (DIA source immunoassays S.A, Belgium) is a solid phase enzyme amplified sensitivity immunoassay. Samples and control were added on a microtitre plate (Figure 5).

**Figure 6:**
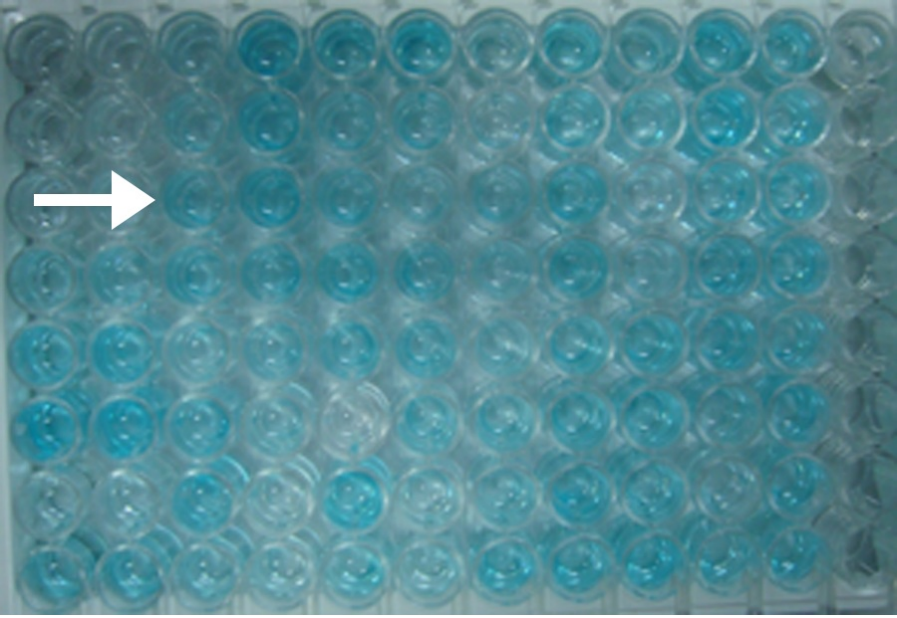
Figure showing the change in the color of the solution in which interleukin-one beta (IL-1 β) has been detected

The plate was incubated for 15 minutes at room temperature on a horizontal shaker set at 700 rpm, 50 µl of the stop solution (to stop the reaction) was pipetted into each well. The absorbencies were read at 450 nm and 490 nm within three hours. There was a change in the colour of the sample in which IL-1β was detected (Figure 6).

The results of the collected samples were calculated using the enzyme-linked immuno sorbent assay - analysis in detail ( ELISA-AIDTM ) technique which processes the data. The plate was first read at 450 nm against a reference filter set at 650 nm. A second reading was performed at 490 nm against the same reference filter. The ELISA-AIDTM technique drives the reader automatically and integrates both readings into a polychromatic model.

The collected data were analyzed with the International Bibliography of Social Sciences. Statistical Package for the Social Sciences (IBSS.SPSS) statistics software 23.0 version (IBM Corp, Armonk, New York, United States). Descriptive statistics mean & standard deviation were used. The normality of the data was verified with Shapiro Wilk's test which showed the data normal. Hence, for the multivariate analysis the one-way analysis of variance (ANOVA ) with Tukey's Post-Hoc test was used and for repeated measures, the repeated measures of ANOVA with Bonferroni test was used. In both the above statistical tools, the probability value 0.05 was considered as significant level.

## Results

All assay procedures were carried out using manufacturer’s instructions. The total IL-1β in the sample was determined in picograms (pg) and the calculation of the IL-1β concentration in each sample was performed by dividing the amount of IL-1β by the volume of the sample.

Table 2 shows the comparison between the materials on day zero, 45th day and 90th day and was highly statistically significant with P=0.0005. The multiple comparisons were done using analysis of variance (ANOVA).

**Table 2:** Multiple comparisons using one way analysis of variance (ANOVA)

Multiple comparisons of one-way ANOVA					
		Mean	S.D	F- value	P-value
0 day	Ceramic	109.63	14.49	24.976	0.0005 **
	Metal	135.29	18.63		
	Zirconia	86.57	12.52		
45 day	Ceramic	106.80	13.17	24.492	0.0005 **
	Metal	133.54	18.89		
	Zirconia	87.54	11.10		
90 day	Ceramic	102.25	13.21	26.285	0.0005 **
	Metal	141.98	27.72		
	Zirconia	79.88	13.66		
** Highly statistical significant at P < 0.001 level					

Table 3 shows the multiple comparison using Tukey's HSD post-hoc test between the materials on day zero, 45th day and 90th day and was highly statistically significant with P=0.0005 except for ceramic & zirconia which were significant at P=0.04 at the 90th day.

**Table 3:** Pairwise comparison using TUKEY’S honest, significant, difference (HSD) post-hoc test

Pairwise comparison using Tukey's HSD post-hoc test			P-value
0 day	Ceramic	Metal	0.003 **
		Zirconia	0.007 **
	Metal	Zirconia	0.0005 **
45 day	Ceramic	Metal	0.001 **
		Zirconia	0.019 **
	Metal	Zirconia	0.0005 **
90 day	Ceramic	Metal	0.0005 **
		Zirconia	0.040 *
	Metal	Zirconia	0.0005 **
** Highly significant at P < 0.01 & * Significant at P < 0.05			

Table 4 shows the multiple comparisons using repeated measure of ANOVA with Bonferroni correction between day zero, 45th day and 90th day. It was found to be statistically significant only for zirconia (p=0.002).

**Table 4:** Multiple comparisons using repeated measures of analysis of variance (ANOVA)

Multiple comparisons using repeated measure of ANOVA with Bonferroni correction					
Material	Days	Mean	S.D	F- value	P-value
Metal	0 day	109.63	14.49	3.008	0.102 #
	45 day	106.80	13.17		
	90 day	102.25	13.21		
Ceramic	0 day	135.29	18.63	1.319	0.292 #
	45 day	133.54	18.89		
	90 day	141.98	27.72		
Zirconia	0 day	86.57	12.52	9.401	0.002 **
	45 day	87.54	11.10		
	90 day	79.88	13.66		
# Not significant at P < 0.05 & ** Highly Significant at P < 0.01					

**Figure 7:**
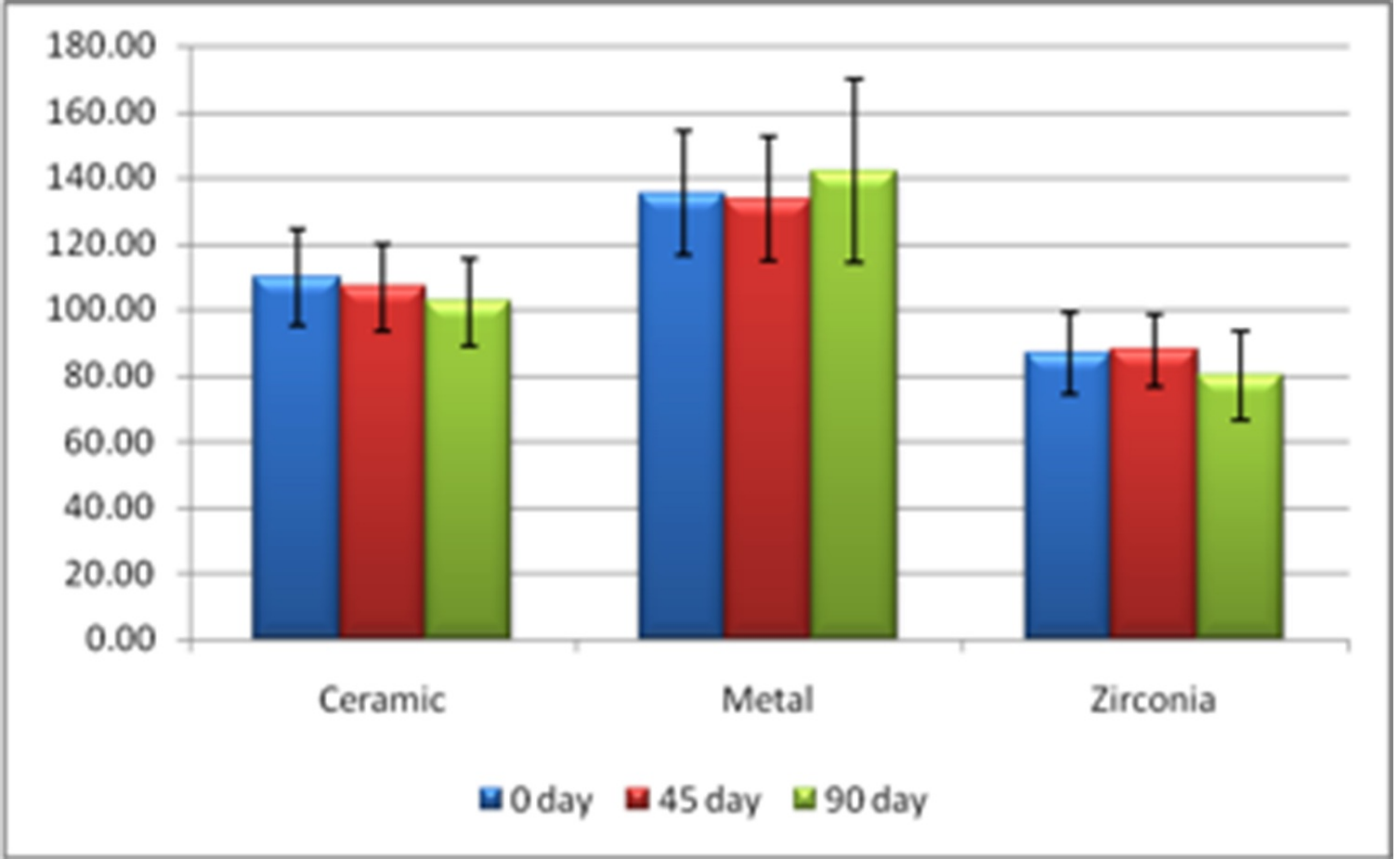
Statistical demonstration of the comparison of various groups in different time periods

Figure 7 gives a comparison between the various groups (metal, ceramic and zirconia) in various time periods. This comparison shows that the zirconia shows the least inflammatory response and is friendly to the oral environment compared to metal or ceramic crowns.

## Discussion

Great efforts through research and clinical trials have been made to achieve the goal of a healthy coexistence between restorations and surrounding periodontal structures. Over the years, many concepts and techniques have evolved and were discarded or modified as they were met with varying degrees of success or failure.

A healthy periodontium, in which the free gingival margin is in a stable relationship to the tooth is essential to the success of a restoration. This healthy periodontium must exist prior to the fabrication of a crown and must be maintained after the crown has been placed. Despite the long-standing use of alloys and ceramics as fixed and removable restoration materials, there are still questions about their behavior in the oral environment [6]. These materials come into close and prolonged contact with gingival and oral mucosa and have been claimed to cause inflammation of these tissues.

Location of margins is the most important aspect in the success of cast restorations [7]. An equi-gingival margin was chosen for this study. The most desirable location of a margin is where the dentist can best control its adaptation and the patient can most effectively clean it [8]. Historically, it was debated that the most desirable location for crown margins is either supragingival or equigingival when possible [9]. Placement of equigingival margins was preferred since the margin will not induce inflammation and bleeding [10]. Marcum found that crown margins at the crest of the gingiva (equi-gingivally) caused less inflammation compared to those below or above the gingival crest [11].

The materials considered in our study were metal, ceramic and zirconia since these materials were economically feasible and widely used for the fabrication of crowns.

Although bleeding on probing is considered a reliable diagnostic criterion in evaluating gingival inflammation [12], it does not give an insight into the biological effects.

Gingival crevicular fluid is particularly attracted as a marker of periodontal disease activity and is a convenient non-invasive and efficient means to sample biomarkers of inflammation and bone resorption in the oral cavity [13] and immune host response in periodontal disease [14]. It is an inflammatory exudate that can be collected at the gingival margin or within the gingival crevice. The collection of GCF may provide information on fluid volume.

Among the numerous cytokines involved in the induction and regulation of host responses in inflammation, IL-1β seems to play a central role in the inflammatory reaction. Kornman, et al. evaluated the importance of interleukin 1-β and its association with inflammatory periodontal disease [15], and showed that an increased production of the gingival crevicular fluid and salivary IL-1β predisposes the patient to chronic periodontitis due to an exaggerated inflammatory response by the immune system.

IL-1β is important in periodontal diseases due to its potency in inhibiting bone formation and enhancing bone resorption stimulating the production of prostaglandin E2, collagenase, and proteinase [16].

Therefore the aim of the study was to evaluate the effect of metal, ceramic and zirconia crowns placed at the level of the gingival crest, on the IL-1β content of GCF.

The first group indicated metal margins of the crown coming in contact with the marginal gingiva. The second group indicated ceramic margins coming in contact with the marginal gingiva. The third group indicated margins of the zirconia crown coming in contact with the marginal gingiva. After crowns were cemented, GCF was collected with the help of periopaper strips from the crowned tooth and the contralateral control tooth.

The IL-1β levels were measured in GCF by comparing tooth with crowns and contralateral natural tooth to monitor the effect of oral hygiene on gingival inflammation. Measurements obtained from contralateral natural teeth revealed no significant differences during the study, so it was concluded that the clinical conditions of the natural tooth and the restored tooth were similar during the study.

The results of our study confirmed that the marginal gingiva in contact with the margins of group one crowns exhibits more inflammation than gingiva coming in contact with the other group of crowns.

The reason for increased gingival inflammation in group one may be due to distortion of the metal substructure that occurs during thermal cycling and a greater mesiodistal opening of the margins in metal ceramic crowns which is not present in all ceramic crowns [17-19]. It is also due to leaching of metal ions coming in contact with marginal gingiva [20-23]. The results of our study were concurrent with the study by Julide Ozen, et al. [20] and proved that metal margins containing nickel-chromium-molybdenum (Ni-Co-Mo) alloy showed higher gingival inflammation compared to ceramic or zirconia crowns.

The mesio-distal opening at the margin leads to more plaque accumulation and in turn increased levels of IL-1β in the GCF. A better overall adaptation of the all-ceramic crowns is one of the reasons for least gingival inflammation in ceramic margins coming in contact with the marginal gingiva [19-23].

No local or systemic adverse reactions or cytotoxic effects of zirconia material have been found [24]. Bacteria and pathogens do not seem to adhere to the zirconia to the same extent as to other materials [25-28].

Heather J.Conrad [29] reviewed the current literature covering all- ceramic materials and the systems which are currently available for clinical use. He stated that the zirconia frameworks with higher elastic modulus were preferred as fixed partial dentures as they reduce the stress on the weaker veneer layer and increase the composite load-bearing capacity, thereby retarding the fracture of the restoration. He also stated that the incidence of gingival inflammation increases around clinically deficient restorations, particularly those with rough surfaces, subgingival finish lines or poor marginal adaptation.

Studies were done by Kirsten [30] for the quantification of inflammatory reaction to ceramic restorations made from lithium disilicate and zirconia by measurement of the concentration of indicators of inflammation in the gingival crevicular fluid (GCF).

### Limitation

 

The GCF samples which were collected from the site with the help of periopaper strips were not measured prior to sampling procedure.

This in vivo study was based on a three-month analysis with 20 subjects. However, the study requires a longitudinal analysis with more number of subjects.

## Conclusions

The marginal gingiva exhibits varied responses when it comes in contact with different types of restorative materials. Studies have also been conducted to prove that crown margins located at the crest of the gingiva caused less inflammation than either those below or above the gingival crest. This study was conducted to evaluate the effect of different crown materials on the amount of inflammation at the marginal gingiva (equigingival margin) by measuring the IL-1β content in the GCF and hence proving the results on a biochemical basis.

At the end of the three-month analysis, it was seen that the zirconia crowns exhibited the least marginal gingival inflammation from the collected GCF samples. The comparison between the three groups (metal, ceramic, and zirconia) was also found to be statistically significant in all the samples (day zero, 45th and 90th day). As no previous studies or literature gives a comparative analysis of the three types of crowns (metal, ceramic, zirconia) on endodontically treated teeth based on IL-1β levels, prosthodontic research, as well as longitudinal studies, must be carried out to further investigate this association.
